# Supplementary material for: Antenatal care utilization and low birth weight in Zambia: Evidence from the Zambia Demographic and Health Survey
Source: PLOS Glob Public Health. 2026 Aug 18;6(8):e0006366. doi: 10.1371/journal.pgph.0006366 (PMC13484962; doi:10.1371/journal.pgph.0006366)
Supplement: Supplementary file 1 [file pgph.0006366.s003.docx]

**Response to Reviewers**

Manuscript ID: PGPH-D-26-01353

**Title: Antenatal Care Utilization and Low Birth Weight in Zambia: Evidence from the Zambia Demographic and Health Survey**

Dear Dr. Kokkinias and Members of the Editorial Board,

We sincerely thank the editor and both reviewers for their careful evaluation of our manuscript and for the constructive comments provided. We have carefully considered each point and have revised the manuscript accordingly. Below, we provide a point-by-point response to each comment. All changes are highlighted in the revised manuscript with track changes.

**REVIEWER 1**

**Comment 1: Exclusion of anaemic women is not justified and likely biases the results. Please include them in a sensitivity analysis or provide strong justification.**

**Response:** We thank the reviewer for raising this important concern. In the original submission, anaemic women were excluded on the basis that anaemia may confound the relationship between ANC attendance and LBW. Upon reflection and in response to this comment, we agree that this exclusion was not sufficiently justified and may have introduced selection bias. We have revised the analysis to include anaemic women in the full analytic sample, treating anaemia as a mediator rather than a confounder. Following this change, the crude association between adequate ANC attendance and LBW was statistically significant (p = 0.025), and the adjusted findings remained consistent with the original results. The manuscript has been updated accordingly throughout the Methods, Results, and Discussion sections.

**Comment 2: Overinterpretation of non-significant findings — the crude OR for ≥8 ANC visits (p = 0.062) is not statistically significant; revise the text accordingly.**

**Response:** We thank the reviewer for this observation. Following the revision described in Comment 1 above — specifically the inclusion of anaemic women in the full sample — the crude OR for ≥8 ANC visits is now statistically significant (p = 0.025). As such, this concern has been addressed through the analytic revision rather than through textual modification alone. The results section has been updated to reflect the revised estimates accurately.

**Comment 3: Statistical reporting in Table 1 is incomplete (tests not specified, p-value discrepancy for province). Correct and clarify.**

**Response:** We thank the reviewer for this observation. Table 1 has been revised to specify the statistical tests used for each variable. The p-value discrepancy for province has been identified and corrected. We have also ensured that all results in Table 1 are survey-weighted, consistent with the complex survey design of the ZDHS.

**Comment 4: Missing model diagnostics (collinearity, goodness of fit, missing data handling) — please report.**

**Response:** We thank the reviewer for this important methodological point. We have now added model diagnostics to the revised manuscript under a new section *“2.3.5 Model diagnostics”.* Multicollinearity among independent variables was assessed using the Variance Inflation Factor (VIF); all VIF values were below 2.0 (range: 1.01–1.83; mean VIF = 1.44), indicating no evidence of multicollinearity. Model goodness of fit was assessed using the Archer-Lemeshow test, which is appropriate for complex survey data; results indicated adequate model fit (F(9, 515) = 0.32, p = 0.969). A calibration plot confirmed close agreement between observed and predicted probabilities across. Missing data were handled through complete case analysis.

**Comment 5: Figure numbering errors — cross-references do not match (e.g., Figure 1 vs. Figure 4).**

**Response:** We apologise for this oversight. All figure numbers and their in-text cross-references have been carefully reviewed and corrected throughout the manuscript to ensure consistency.

**Comment 6: Language and typos — "non anemic", "Mucunga", "vividly decrease" — requires proofreading.**

**Response:** We thank the reviewer for drawing attention to these errors. The manuscript has been thoroughly proofread and all identified typographical and grammatical errors have been corrected, including those specifically noted. We have also undertaken a general language revision of the full manuscript to improve clarity and precision, Professor Choolwe Jacobs, a senior author on the paper reviewed and edited the manuscript.

**REVIEWER 2**

**Comment 1: What percentage of birth weight data was reported by mothers? Was there any validation of these data?**

**Response:** We thank the reviewer for raising this important data quality concern. We have calculated the proportion of birth weight observations derived from maternal recall (1900 (45.5%)) versus child health cards (2278 (54.5%)) and have reported this in the revised Methods section. Regarding validation, the ZDHS follows standardised DHS protocols for birth weight data collection, which include structured interviewer training and quality assurance procedures. We acknowledge that maternal recall may be subject to recall bias and have noted this explicitly as a limitation. To minimise its potential impact, the analysis was restricted to the most recent singleton live birth for each woman, reducing the recall period.

**Comment 2: There is no need to report variable numbers such as v005, v021, and v022. Please clarify whether the logistic regression incorporated survey weights and accounted for stratification.**

**Response:** We thank the reviewer for this suggestion. The variable reference codes (v005, v021, v022) have been removed from the manuscript. Regarding the analytical approach, we confirm that all logistic regression models incorporated survey weights and accounted for both clustering and stratification using the svyset command in Stata version 17, consistent with the complex survey design of the ZDHS. This has been clarified explicitly in the Methods section.

**Comment 3: Was survey year included in the logistic regression model?**

**Response:** We thank the reviewer for this question. The logistic regression analysis was conducted exclusively using data from the 2024 ZDHS round. Survey year was therefore not applicable as a covariate in the regression model, as there was no variation in survey year within the analytic sample. The pooled 2001–2024 data were used solely for the spatial and temporal trend analyses. This distinction has been clarified in the Methods section.

**Comment 4: Were any participants repeatedly surveyed over the years? If more than one participant came from the same household, was any adjustment made for within-household correlation?**

**Response:** We thank the reviewer for this methodological question. The DHS employs a cross-sectional design, and participants are not followed across survey rounds; each round constitutes an independent sample. Within the 2024 ZDHS round used for the regression analysis, within-household correlation was accounted for through the complex survey design, specifically through the specification of primary sampling units (clusters) as the unit of clustering in the svyset command. This ensures that standard errors are appropriately adjusted for the clustered nature of the data.

**Comment 5: Table 1 should present weighted results.**

**Response:** We thank the reviewer for this comment. Table 1 has been revised to present survey-weighted results throughout, consistent with the complex survey design of the ZDHS.

We believe the revised manuscript is substantially strengthened by these revisions and hope it now meets the standards for publication in PLOS Global Public Health. We remain available to provide any further clarification the editorial team may require.

Yours sincerely,

**Mr. Teebeny Zulu**

On behalf of all co-authors
